# Potential of nanoemulsions for accelerated wound healing: innovative strategies

**DOI:** 10.1097/JS9.0000000000000460

**Published:** 2023-05-09

**Authors:** Jatin Chhabra, Hitesh Chopra, Rakesh Pahwa, Neha Raina, Karan Wadhwa, Swati Saini, Poonam Negi, Madhu Gupta, Inderbir Singh, Harish Dureja, Talha Bin Emran

**Affiliations:** a Institute of Pharmaceutical Sciences, Kurukshetra University, Kurukshetra; b Department of Pharmaceutical Sciences, Maharshi Dayanand University, Rohtak, Haryana; c Department of Pharmaceutics, Delhi Pharmaceutical Sciences & Research University, New Delhi; d Chitkara College of Pharmacy, Chitkara University, Punjab, India; e School of Pharmaceutical Sciences, Shoolini University, Solan, Himachal Pradesh, India; f Department of Pharmacy, BGC Trust University Bangladesh, Chittagong, Bangladesh; g Department of Pharmacy, Faculty of Allied Health Sciences, Daffodil International University, Dhaka, Bangladesh

**Keywords:** futuristic developments, mechanistic insights, nanoemulsions, nanoemulsions based hydrogels, natural oils, wound healing

## Abstract

Wounds represent various significant health concerns for patients and also contribute major costs to healthcare systems. Wound healing comprises of overlapped and various coordinated steps such as homeostasis, inflammation, proliferation, and remodeling. In response to the failure of many strategies in delivering intended results including wound closure, fluid loss control, and exhibiting properties such as durability, targeted delivery, accelerated action, along with histocompatibility, numerous nanotechnological advances have been introduced. To understand the magnitude of wound therapy, this systematic and updated review discussing the effectiveness of nanoemulsions has been undertaken. This review portrays mechanisms associated with wound healing, factors for delayed wound healing, and various technologies utilized to treat wounds effectively. While many strategies are available, nanoemulsions have attracted the tremendous attention of scientists globally for the research in wound therapy due to their long-term thermodynamic stability and bioavailability. Nanoemulsions not only aid in tissue repair, but are also considered as an excellent delivery system for various synthetic and natural actives. Nanotechnology provides several pivotal benefits in wound healing, including improved skin permeation, controlled release, and stimulation of fibroblast cell proliferation. The significant role of nanoemulsions in improved wound healing along with their preparation techniques has also been highlighted with special emphasis on mechanistic insights. This article illustrates recent research advancements for the utilization of nanoemulsions in wound treatment. An adequate literature search has been conducted using the keywords ‘Nanoemulsions in wound healing’, ‘Wound therapy and nanoemulsions’, ‘Herbal actives in wound therapy’, ‘Natural oils and wounds treatment’ etc., from PubMed, Science Direct, and Google Scholar databases. Referred and original publications in the English language accessed till April 2022 has been included, whereas nonEnglish language papers, unpublished data, and nonoriginal papers were excluded from the study.

## Introduction

Highlights

- Wounds represent various significant health concerns for patients.
- This review portrays mechanisms associated with wound healing.
- Nanotechnology provides several pivotal benefits in wound healing.
- This review focuses on advancements for the utilization of nanoemulsions in wound treatment.

The skin’s primary function is to act as a defensive barrier against environmental intrusion, including sun radiation and air pollution. When the structural integrity of the skin is compromised, the immune system is impacted, which may lead to morbidity and mortality^1^. Skin repair and regeneration is a complicated, multifaceted process that involves a highly sophisticated temporal sequence of molecular mechanisms and cellular processes that are triggered by injury and continue sequentially and in harmonious manner to heal the injured tissue^2^.

**Figure 1:**
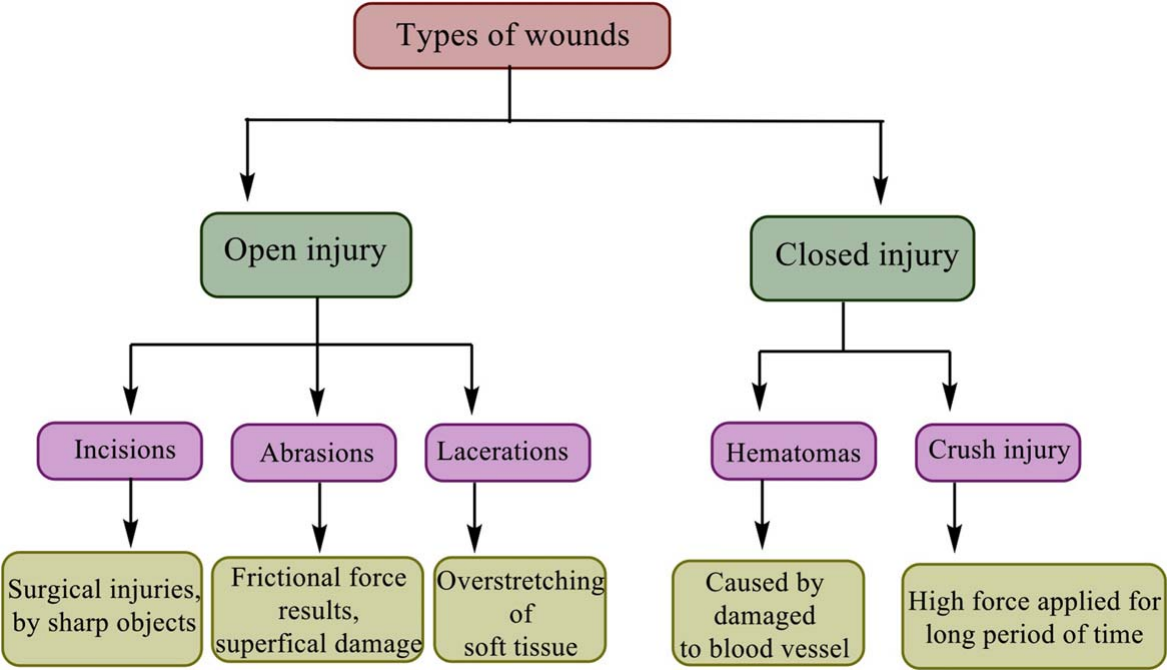
Classification of wounds.

A wound is an injury or distortion to physiological structure and functions caused by a simple or significant break in the anatomy of an organ such as the skin, which can spread to nearby tissues and structures including subcutaneous tissue, muscles, tendons, nerves, and arteries, among others^3^. The microbiota of the skin is intricately related to skin health and illness. The immune response is regulated, and barrier restoration is promoted by interaction between commensal bacteria and various cell types engaged in cutaneous wound healing. This conversation between host cells and the microbiota is disturbed in wounds^4^. Wounds are generally classified into two types, such as acute injury and chronic injury based on their healing time. Acute injuries heal in a predictable time, followed by structural and functional tissue restoration. While, chronic injuries do not heal within a reasonable time frame, resulting in additional microbial infection complications and difficulty in healing^5^. In a similar manner, wounds are categorized as either open (cutaneous) or closed (noncutaneous) injuries. An open injury is sometimes defined as a surgical incision (cut) and is further classified as abrasions (superficial injury) and lacerations (frictional force). When an injury is under the skin and not exposed to the air, it is called a closed injury. Closed injuries can be classified as hematomas (blood vessel disruption) and crush injuries (high-pressure) (Fig. 1). Various microorganisms found in wounds including gram-positive, gram-negative, and fungi are described in Table 1.

**Table 1:** Microorganisms found in wound infection^6–8^.

Group	Species
Gram-negative	*Acinetobacter, Enterobacter Cloacae, Escherichia Coli, Proteus Mirabilis, Serratia Marcescens, Klebsiella Pneumoniae*
Gram-positive	*Enterococcus Avium, Staphylococcus Aureus, Staphylococcus Haemolyticus, Staphylococcus Schleiferi, Enterococcus Faecalis*
Fungi	*Candida Albicans, Candida Glabrata, Candida Stellatoidea, Candida Tropicalis*

### Wound healing and its phases

**Figure 2:**
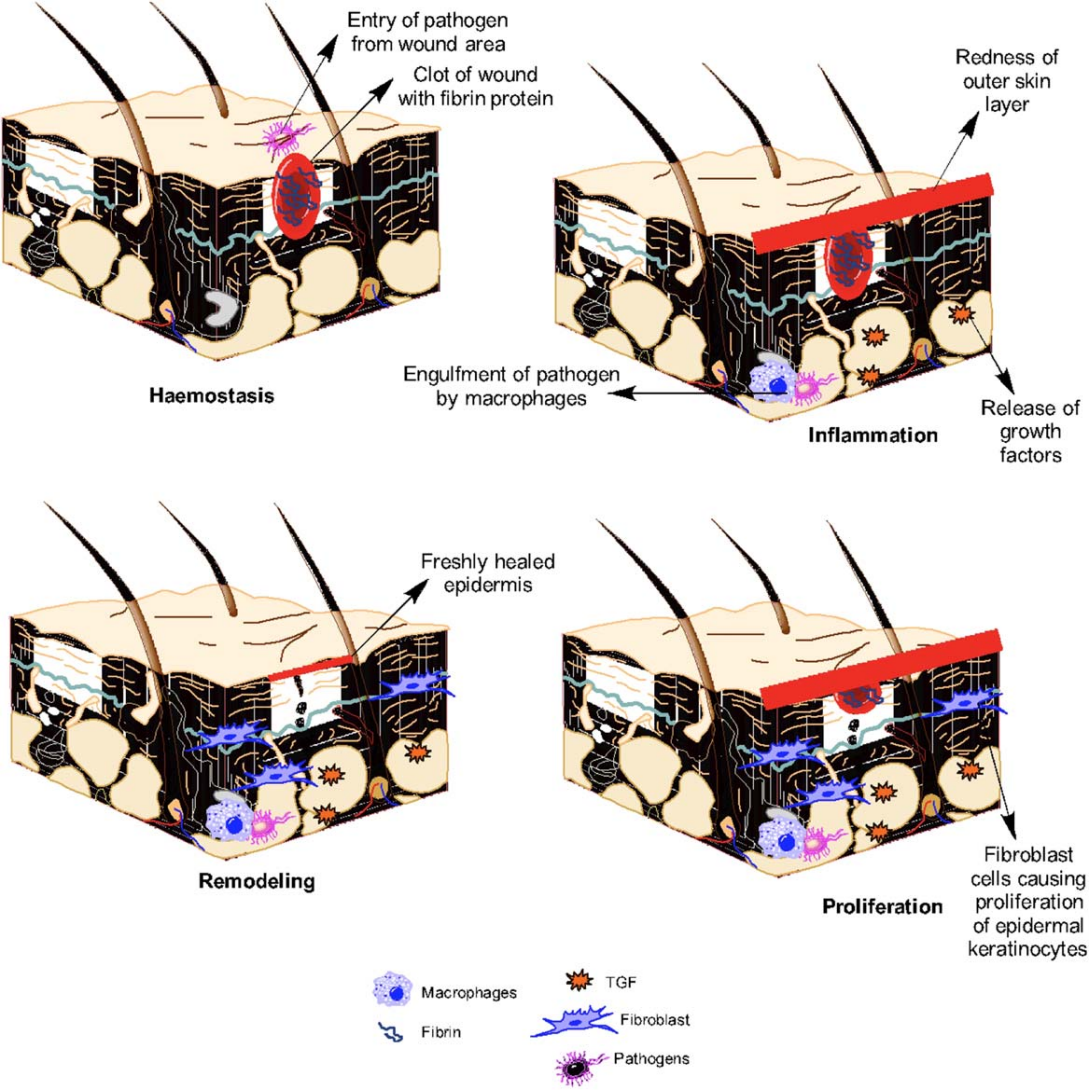
Mechanism of wound healing.

Wound healing is a multi-step process, which begins with an injury. This injury response is a phylogenetically primal, yet necessary innate host immune response, for tissue integral restoration^9^. The entire process is usually classified into four stages (Fig. 2) including hemostasis, inflammation, proliferation, and remodeling^10,11^. Hemostasis begins just after an injury^12^, causes vessel rupture, exposure of subendothelial collagen to platelets, thereby causing accumulation and activation of the coagulation cascade. Afterwards, neutrophils and then macrophages travel to the site of injury, resulting in the initiation of the inflammation phase, via the production of inflammatory mediators such as interleukin-4 and transforming growth factor (TGF-β). The primary goal of the inflammatory phase is to keep the wound free from becoming infected and persists until all excess bacteria and debris from the wound are removed^13,14^.

**Figure 3:**
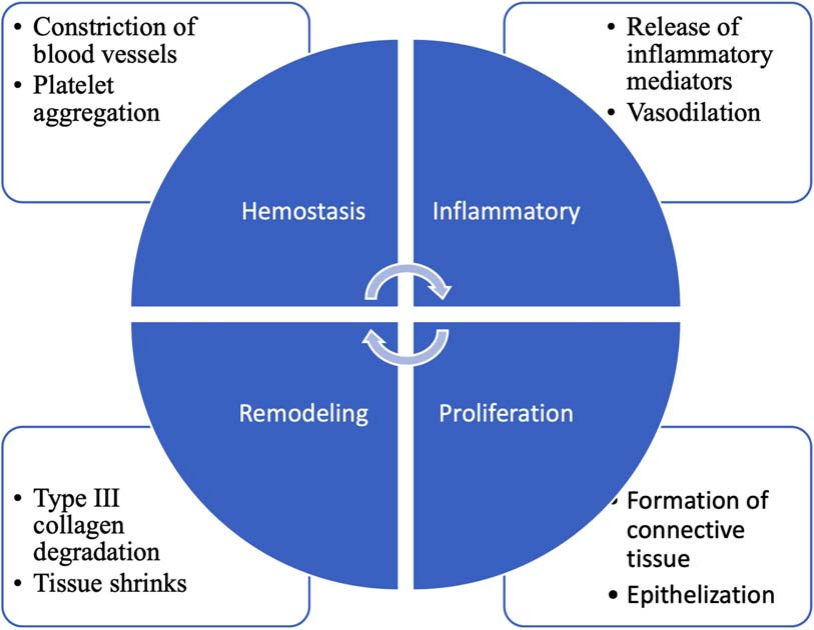
Phases of wound healing and the events involved.

The proliferative phase, which is characterized by fibroblast migration, extracellular matrix deposition, and the formation of granulation tissue, begins around day 3 and continues for 2–4 weeks after wounding. In this phase, fibroplasia and angiogenesis occur synchronously and in a tightly coordinated manner to form extracellular matrix and granulation tissue^15,16^. The final step in the proliferation phase is re-epithelialization, which entails numerous steps, *viz*, the migration of neighboring epidermal keratinocytes into the wound, the proliferation of keratinocytes, the development of neoepithelium into the stratified epidermis, as well as the restoration of an integral basement membrane region that links the epidermis to the underlying dermis. The process of re-epithelialization can only begin once the wound surface is appropriately established with proliferating fibroblasts, new vessels, and collagen matrix^17,18^. The final phase of wound healing, that is, maturation or remodeling is approximately the same in all wounds and lasts for months or even years^19^. Collagen III from the newly produced extracellular matrix is eventually replaced by collagen I, which reveals an extra organized lattice structure and improves the tensile strength of the repaired skin^20^. Matrix metalloproteinases, which are produced by macrophages, neutrophils, and fibroblasts in the wound are accountable for collagen degradation. The underlying connective tissue shrinks, causing the wound borders to become closer together. Capillary growth reduces blood supply and hence metabolic activity, resulting in the production of a developed scar with fewer cells and a high tensile strength^21^. The key events involved in all four phases are summarized in Fig. 3.

### Factors influencing wound healing

There are numerous local and systemic elements that delay wound healing. Examples of local factors include tissue hypoxia^22–24^, infection^25–27^, and hydration^28–30^. Systemic factors include ageing^31,32^, smoking^33–35^, stress^36,37^, diabetes^38^, and obesity^39^. To reduce or manage the influence of these factors, different strategies for wound treatment are available like debridement^40^, negative pressure therapy^41^, oxygen therapy to provide supplement oxygen^42^, and gene therapy^43^. Autografts and allografts are commonly used techniques that effectively generate fascia with full thickness from the donor site of patients or other donators and graft it in the required zone^44^. The use and administration of growth factors like platelet-derived, fibroblast is considered to be essential for the onset and management of the wound healing process and can also consider as a significant therapeutic substitute for healing wounds^45^. Aside from the above-described techniques, different strategies like electrical stimulation^46^, induced pluripotent stem cell^47^, and low-level laser therapy^48^ that enhance wound healing activity are also available.

### Salient dosage forms for wound healing

**Figure 4:**
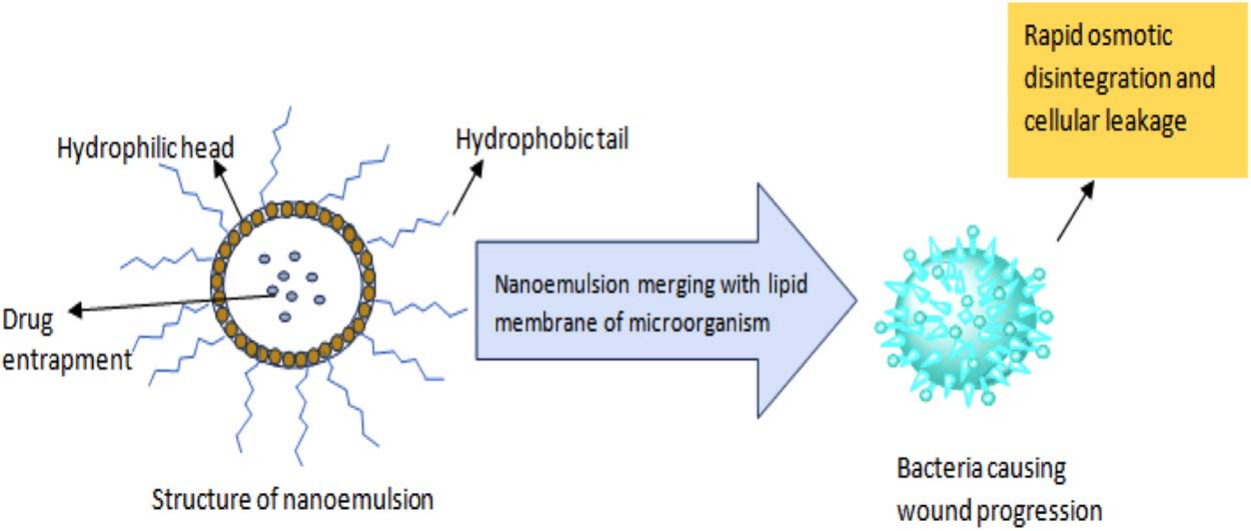
Possible mode of action of nanoemulsions against wound microorganisms.

**Figure 5:**
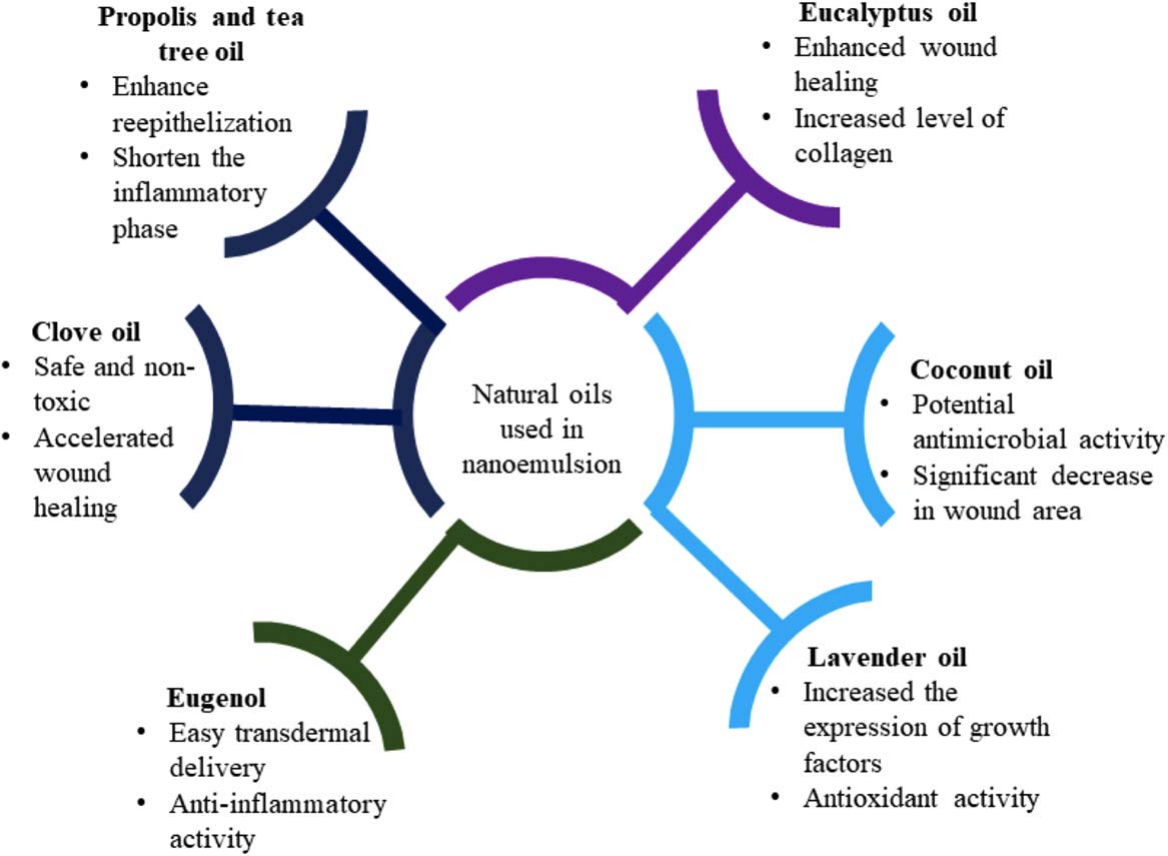
Different natural oils for preparation of nanoemulsion and their significant effect on wound healing.

Different scientific studies reported important dosage forms like hydrogels^49–54^, electrospun membranes^55–57^, wafers^58–61^, nanoparticles^62–64^, nanoemulsions^65^, and nanoemulsion-based hydrogels^66–68^ used for targeted delivery of drugs against wounds. Nanotechnology-based treatment methods provide an excellent and fascinating opportunity to achieve targeted action towards wounds. Diagnostics and treatment approaches based on nanotechnology provide an overview to target the heterogenicity of the wound healing process. Among the dosage forms listed, nanoemulsion is a promising nanocarrier with huge and potential applications in wound healing^69–71^ and also offers numerous benefits, *viz.* enhanced permeability, controlled drug delivery, higher stability, targeted action, and high binding interaction with the lipid layer of the skin. The use of nanoemulsions can improve antibacterial efficacy along with reduced droplet size dispersion causing accelerated wound healing. Moreover, nanoemulsions also have the ability to increase the solubility of drug, resulting in high therapeutic efficacy against wounds. Bacteria and viruses causing wound progression interacts with the lipid layer of nanoemulsions resulting cell wall disruption and subsequently leakage of cellular content. The possible mode of action of nanoemulsions against wound microorganisms is illustrated in Figure 4 ^72,73^. It has been found that the oily phase of nanoemulsions affects their wound healing properties^74^. Propolis oil containing flavonoids and caffeic acid significantly reduced the inflammatory phase by inhibiting lipoxygenase activity resulting in reduced production of prostaglandins, which further stimulate the immune cells. Moreover, oils can also accelerate debriding activity triggering faster wound healing^75^. Eucalyptus oil and clove oil significantly enhanced the production of collagen and leucine, respectively, resulting into accelerated wound healing^76,77^. Different natural oils and their role in wound healing are illustrated in Figure 5.

### Advantages of nanoemulsions in wound healing

The emergence of innovative therapies in the field of wound healing highlights the potential role of nanotechnology-based drug delivery systems as a viable approach to promote healing mechanisms at various stages. Nanoemulsions have been affirmed to be an superior choice because of their several properties which resulted in their enlarge utilization in wound therapy. The main benefits of using nanoemulsions as drug delivery carriers in the treatment and management of wound healing include high drug loading capacity, improved drug solubility, and bioavailability, relatively simple preparation and scale-up, controlled drug release, and protection from enzymatic degradation. By lowering surface and interfacial tension and so elevating the system’s overall viscosity and spreadability, the nanoemulsion gives the system thermodynamic stability. Because of its high drug loading capacity, improved difusibility, permeability, and reduced skin irritation, nanoemulgel has several benefits over lipid nanoparticles, microemulsions, or liposomes in transdermal or dental delivery^78–80^. The numerous benefits of nanoemulsions for enhancing wound healing prospects are also summarized in Table 2.

**Table 2:** Important advantages of nanoemulsions in wound therapy.

Advantages	References
Protection of drug from hydrolysis and oxidation	^76^
Controlled drug delivery at wound site	^78^
Higher bioavailability resulted in rapid absorption	^78^
Easy penetration into skin layer and uniform dispersion	^79^
Accelerate wound healing process	^80^

### Preparation methodologies

**Figure 6:**
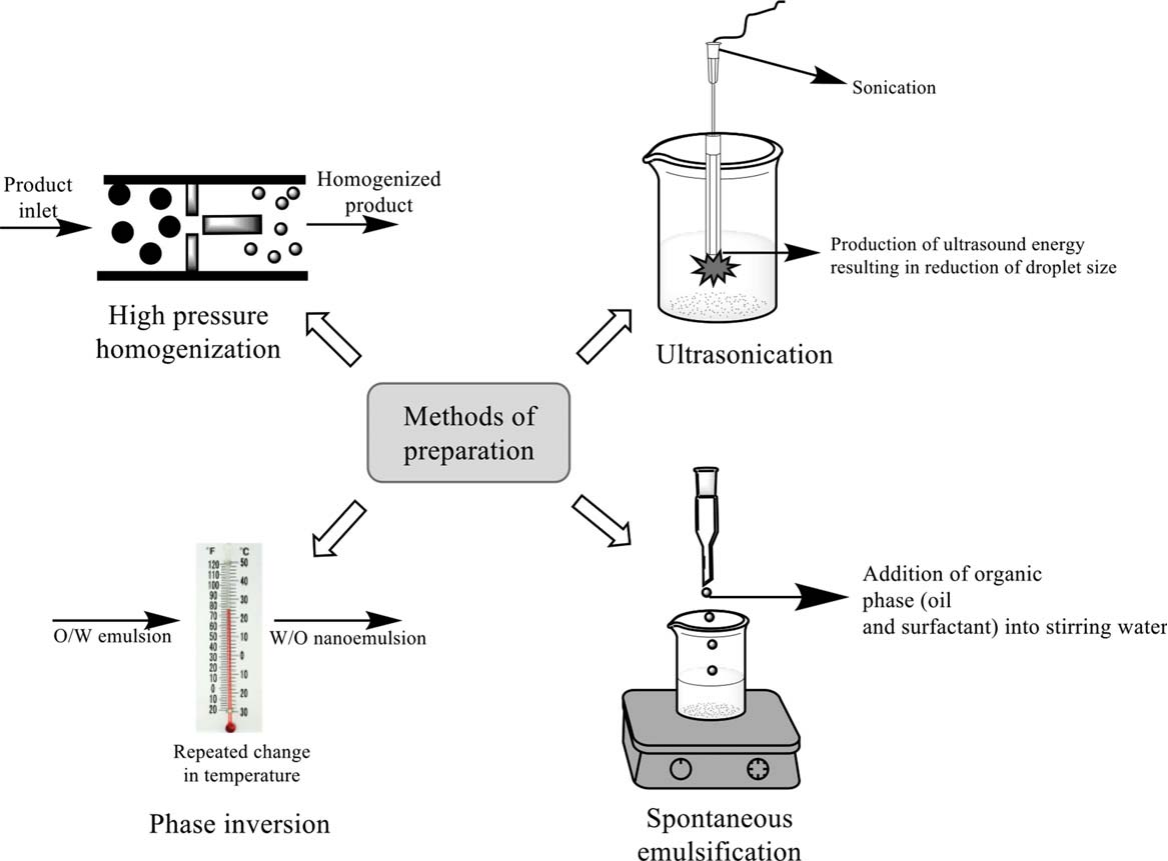
Some important techniques utilized for the preparation of nanoemulsions for wound therapy.

Various preparation methods of nanoemulsion for wound therapy are broadly classified into two categories as: high energy and low energy approaches. High energy methods concerning with wound healing are further classified as high-pressure homogenization and ultrasonication. Whereas low energy methods include phase inversion and spontaneous emulsification^81,82^. Some suitable techniques utilized for the nanoemulsion preparations in wound therapy are illustrated in Figure 6.

#### High energy methods

Large disruptive energies are provided using mechanical forces in the high energy approach. The droplet size depends on the equipment, preparation parameters such as temperature and time, as well as the characteristics of the sample. High energy methods are complex and energy-intensive making them expensive, thus, this method is not suitable for thermosensitive products^83^. This method is divided into the following categories as: high-pressure homogenization and ultrasonication.

#### High-pressure homogenization

This technique is frequently employed for the development of nanoemulsions using various forces, *viz*. intense turbulence, cavitations, and hydraulic shear, and the particle size of approximately 1 nm can be produced with this system. It utilizes a high-pressure piston/ homogenizer in which two liquids comprising of surfactants and co-surfactants are pushed under intense pressure (500–5000 psi) through a small orifice to distort the interface between the water and oil to such an extent that the formed droplets are broken up into smaller one. Indeed, the development of nanoemulsions through the high-pressure homogenization method is not impulsive in nature, as it necessitates force input either in the form of chemical or mechanical. Furthermore, if the system’s temperature rises during processing, the preparation may become unstable^83,84^. This technique has wide applications in wound therapy utilizing herbal actives *viz.* betulin-enriched extract, oil palm (*Elaeis guineensis Jacq*.) leaf, etc. Emulsification from this technique leads to a significant reduction in droplet size, which further influence the targeted delivery of drug. Moreover, optimum drug loading and polydispersity index was also observed^85,86^.

#### Ultrasonication

Nanoemulsion can be easily fabricated using ultrasonication technique via the agitation of molecules trigged by sound waves that later on break oil droplets through local turbulence. A probe, a metal horn, a generator (which produces electric waves), and a piezoelectric transducer are typically used to produce nanoemulsions using ultrasonication^86^. In contrast to the high-pressure homogenization method, it requires less energy, easy manipulation, and cost effective. The energy is delivered using sonicator probes, which are sonotrodes. It includes a piezoelectric quartz crystal, which expands and contracts in response to alternating electric energy. When the sonicator’s tip makes contact with the liquid, it causes mechanical vibration and cavitation. Despite its enormous ability, the approach is still limited to laboratory studies and only pertinent for pilot-scale fabrication because of the influence of the wave around the molecules that can affect large-scale production^84,87,88^. Various synthetic (clindamycin) as well as herbal constituents (eugenol) nanoemulsions have been prepared by this method and showed effective wound healing. A high skin disposition with optimal viscosity was achieved. Moreover, Transmission Electron Microscopy analysis also showed that the nanoemulsions formed had spherical and reduced droplet size^75,89^.

#### Low energy methods

Low energy techniques use the characteristics of the surfactant, oil, and water system and also do not require any specific equipment. Because of their low cost and ease of implementation, low energy technologies have prompted a surge in a nanoemulsion study for their development and enormous applications^84^. Low energy emulsification techniques use the system’s intrinsic chemical energy and only mild agitation to fabricate nanoemulsions, making them more energy-efficient. This method can be classified as phase inversion and spontaneous emulsification.

#### Phase inversion

The phase invasion technique fabricates nanoemulsion via altering the surfactant’s spontaneous curvature. Under this technique, the utilization of nonionic surfactants alters the system’s temperature, enabling an oil-in-water emulsion (O/W) to transform into a water-in-oil emulsion (W/O) at higher temperatures. For phase inversion emulsification, a critical surfactant concentration is required. Furthermore, the elevation in temperature dehydrates the polymeric chain of surfactant, making it more lipophilic; whereas, it forms a large positive spontaneous curvature at low temperature. Thus, sufficient phase transitions can only be produced by altering the composition at a steady temperature or by changing the temperature at a stable composition^84,90^. Besides, in some instances, high energy input is usually required to fabricate micrometer ranged droplet sizes via the phase inversion approach, which can be accomplished by high shear stirring or ultrasound generators. This method has wide applications in wound dressings for the delivery of agents like phenytoin and chlorhexidine. Formulation prepared by this technique was found to be stable having high monodispersity. Enhanced drug entrapment efficacy with no sign of precipitation was observed. Moreover, nanoemulsions prepared by this method revealed enhanced wound healing and provided targeted action^91^.

#### Spontaneous emulsification

This method is also known as self-emulsification. Droplet formation can be achieved by contacting two immiscible liquid that are not in equilibrium without the need of external energy input. One of the foremost advantages of this technique is that it does not need any particular equipment for the fabrication of nanoemulsion and can be developed effectively at room temperature. However, the presence of solvent and the formation of a less oil-phase are two major drawbacks of the self-emulsification technique. Indeed, this method has wide applications in developing herbal actives loaded nanoemulsions for wound healing. Stable and transparent nanoemulsions were achieved after 30 min of sonication. The droplet size obtained had a large surface area resulting in improved absorption through the skin. Quantitative analysis of nanoemulsions in ultraviolet and visible wavelength showed decreased absorbance with an increase in sonication time^65,92,93^.

### Recent investigational studies on nanoemulsions for wound therapy

Nanoemulsions are considered as rational and impressive dosage form for the effective delivery of medicinal agents at the target region in a safe and efficient manner. Significant research has been done globally on nanoemulsions as carriers of wound healing agents. Nanoemulsion formulations for wound healing have been found to reveal inhibitory and stimulatory effects on inflammatory cytokines and antioxidant enzymes, respectively^76^. Back *et al.* fabricated an isoflavone-rich nanoemulsion that increased keratocyte proliferation and migration. A study revealed that nanoemulsions boosted the wound healing process by promoting angiogenesis, reducing lipid oxidation and inflammation, while enhancing re-epithelialization^94^. Moreover, wounds treated with nanoemulsions displayed a higher level of collagen resulting in faster wound healing^95^. Eucalyptus oil nanoemulsions prepared by Sugumar *et al.*, showed potent antibacterial activity against *Staphylococcus aureus.* When tested against animal, nanoemulsion exhibited potential wound healing activity (100% wound closure on day 16^th^) with no sign of skin irritancy^93^. Nanoemulsions for wound healing also significantly enhanced the proliferation of fibroblast cells resulting in a reduction in the time of wound closure^65^. In addition to wound healing effect, insulin loaded nanoemulsions also helped in mitigating diabetes^96^. Lavender oil nanoemulsions prepared by Kazemi *et al.*, effectively reduced the wound area and significantly increased TGF β1, Type I, and Type II collagen synthesis. Additionally, prepared formulations also displayed the antioxidant potential of superoxide dismutase and glutathione peroxidase resulting in reduced MDA level and lipid peroxidation^97^. Zain *et al.*, developed nanoemulsions using flavonoid-rich oil palm (*Elaeis guineensis Jacq.)* leaf extract. Furthermore, wound healing activity and toxicity was evaluated using three different models such as 3T3 fibroblast cells, embryonic and adult Zebrafish. However, the extract and its nanoemulsion were found to be more sensitive towards Zebrafish model. LC_50_ value of the developed nanoemulsions was observed to be higher than that of unloaded leaf extract. Prepared nanoemulsions displayed accelerated wound healing properties when compared with oil palm leaf extract. Additionally, the inhibitory and stimulatory effects of nanoemulsions on inflammatory cytokines and antioxidant enzymes, respectively, suggested that it has strong potential in wound management^86^. Farahani *et al.*, prepared a nonfibrous formulation using cellulose acetate and gelatin (CA/GL). Furthermore, a nanoemulsion of *Zataria multiflora* (antibacterial plant) was also fabricated into a nonfibrous mat. By increasing the CA/GL ratio, improved mechanical strength of the developed formulation was observed. Prepared formulations showed potential activity against *E. coli* and *S. aureus*. *In vivo* evaluation of the nonfibrous formulation showed an accelerated wound healing profile^73^. Nanoemulsions preparations of different drugs have also been summarized in Tables 3 and 4 as synthetic molecules and natural actives, respectively.

**Table 3:** Synthetic molecules loaded nanoemulsions for wound healing.

Sl. No.	Drug	Methods of preparation	Major additives	Droplet size	Zeta potential	Model used	Salient features	References
1.	Clindamycin hydrochloride	Ultrasonication (O/W)	Propolis and tea tree oil.	19.42±1. 7 nm	−24.5±0.2 mV	Human skin fibroblast.	Improved re-epithelialization, collagen synthesis and potent anti-inflammatory action.	^75^
2.	Levofloxacin	Low energy method	Sesame oil, Tween 80, Tween 85.	18±5 nm	0.271 mV	L929 mouse fibroblast cells	Significant reduction in period of epithelialization, wound contraction, and number of inflammatory cells.	^95^
3.	Chlorhexidine acetate	Phase inversion method.	Tween 80, PEG	6.13 nm	−67.13 mV	MRSA cells	Higher activity towards MRSA.	^72^
4.	Phenytoin	Phase inversion method.	Alkyds and Tween 80 surfactant.	11.7±0.07 to 13.5±0.13 nm	−7.0±0.04 to −7.9±0.69 mV	Human keratinocytes (HaCaT cells)	Enhanced proliferation of epidermal cells.	^91^
5.	Insulin	–	Oleic acid, Tween 80, PEG 400.	458±132 nm	−10.2±3.87 mV	Wistar rats	Synergistic effects towards wound healing.	^96^
6.	Simvastatin	Ultrasonication method.	Coconut oil, surfactant, co- surfactant.	186.0±2.5 nm	20±1.2 mV	–	6-fold increased inhibition zone against *Staphylococcus aureus*, synergistic effects.	^98^

**Table 4:** Natural actives loaded nanoemulsions for wound healing.

Sl. No	Drug	Methods of preparation	Major additives	Droplet size	Zeta potential	Model used	Salient features	References
1.	Betulin-enriched extract and purified spruce balm	High-pressure homogenization	Sunflower oil, Jojoba oil, Triglycerides	153 to 245 nm	−67.58 to −77.13 mV	Human keratinocytes and fibroblast cells	Enhanced proliferative profile of keratinocytes and fibroblasts.	^85^
2.	Oil palm (*Elaeis guineensis Jacq*.) leaf	High-pressure homogenization	Palm oil and Gelatin	<100 nm	−24.03 to −34.10 mV	3T3 fibroblast cells, embryonic and adult zebrafish.	Improved and better wound healing potential.	^86^
3.	Astaxanthin-alpha tocopherol	Spontaneous emulsification method, ultrasonication method.	Span 80, Tween 20, PEG	189 to 216.6 nm	−20.57 mV	T24, Panc1, CT26, HeLa	Faster healing effect of nanoemulsion and significant antibacterial activity.	^65^
4.	*Zataria multiflora* essential oil	Ultrasonic homogenization, electrospinning technique.	Cellulose acetate, Glacial acetic acid, Gelatin.	79. 1 nm	–	L929 mouse fibroblast cells	Promoted the adhesion and proliferation of L929 fibroblast cells significantly.	^73^
5.	Eugenol	Ultrasonication method	Tween 80, Larbasol	89.98±6.48 nm	−10.05±0.11 mV	Wistar rat hind paw edema mode	High wound healing and easy transdermal delivery.	^89^
6.	Lavender essential oil and liquorice extract.	Spontaneous emulsification.	Tween 80, Tween 20, Glycerin, PEG 400.	–	–	Wistar rats	Potential wound healing effect.	^97^
7.	Tetrahydroxy curcumin derivative.	High-pressure homogenization.	Tween 10 and Lauroglycol 90, Labrafac PG.	100–300 nm	−30.1 to −31.1 mV	Rat skin	Displayed optimistic results against both gram-positive and negative bacteria.	^99^
8.	Curcumin	–	Labrafac PG, Triacetin, Tween 80, PEG 400.	84.032±0.023 nm	–	Albino rats	Enhanced skin permeation.	^100^
9.	Isoflavones aglycones	Spontaneous emulsification method.	Daidzein, Glycitein, Genistein, Egg lecithin.	50 nm	−60 mV	Wistar rats	Increased reepithelization and angiogenesis rate to promote wound healing.	^94^
10.	Alpha tocopherol	Spontaneous emulsification method.	Chitosan and oleic acid.	220 nm	−56.8±2.1 mV	Human skin cells	Improved alpha tocopherol stability due to encapsulation.	^92^
11.	Eucalyptus oil	Ultrasonic emulsification.	Eucalyptus oil and Tween 80.	3.8 nm	–	Wistar rats	Complete loss of viability within 15 min.	^93^
12.	Naringenin	Low energy emulsification technique.	PEG 400, Tween 20, Tween 80, Chitosan, Lauroglycol 90 and Capryol 90.	15.69±0.737 nm	−8.33±3.09 mV	NHT-3T3 mouse cells	Accelerate wound healing.	^101^
13.	Eucalyptus oil	Aqueous phase titration.	Diethylene glycol monoethyl ether, Ethanol, Eucalyptus oil, Polyoxyethylene sorbitan trioleate.	32.45 nm	−34.25 mV	Rat	Safe, nontoxic and rapid wound healing.	^77^
14.	Clove oil	Spontaneous emulsification method.	Clove oil, Triacetin, Tween 80.	29.10 nm	−31.40 mV	Rat	Significant proliferation of epithelial cells.	^76^

Therefore, major and ongoing research efforts have been made around the world towards developing effective nanoemulsions for accelerated wound healing. Treatment of wounds using nanoemulsion technology showed various significant benefits like targeted, effective, and faster wound healing potential. Also, very few patents are available discussing nanoemulsions for wound healing. Therefore, the patent segment still requires significant attention towards the application of nanoemulsions in wound healing. Some patents on nanoemulsions for wound healing are discussed in the subsequent section.

Notably, in 2016, a patent (US 9,259,407 B2) was published describing the composition and therapeutic usage of nanoemulsions in wound treatment, respiratory infection, bacterial infection, and immunogenic composition-related immune response. In addition, the patent also provided information about clinical, industrial, and research applications^102^. Nanoemulsion’s involvement in the prevention and treatment of burn wounds is also described in the US patent (US 2019 / 0021998 A1) published in 2019. The Patent also detailed the accelerated wound healing activity of nanoemulsions. Moreover, therapeutic uses with clinical, commercial, and scientific applications are also highlighted^103^. Undoubtedly, plant-based bio-active compounds are decidedly sought-after active ingredients in the cosmeceutical and pharmaceutical industries, because of their bounteous therapeutic potentials, and are widely used in the development of nanotechnology-based drug delivery^104^. Several patents have been filed worldwide utilizing plant-based bio-active incorporating in nanoemulsion for the treatment of wounds. Kaur and kapoor^105^, fabricated *Hippophae rhamnoide* (Sea Buckthorn) seed oil incorporated nanoemulsion gel for wound healing. Similarly, Dubey *et al.* ^106^, also filed a patent for topical nanoemulgel containing *Moringa oleifera* seed oil using a low energy emulsification technique for wound therapy.

To further enhance the wound healing applications, different research groups have also focused on nanoemulsion-based hydrogels resulting in increased viscosity of the formulation, which ultimately improved the retention time of the drug. Different nanoemulsion-based hydrogels formulations for wound healing are summarized in Table 5.

**Table 5:** Nanoemulsion based hydrogels preparations for wound healing.

Sl. No.	Drug	Method	Additives	Droplet size	Zeta potential	Model used	Salien t features	References
1.	Thymoquinone	High energy emulsification technique.	Oleic acid, isopropyl myristate, isopropyl alcohol, ethyl oleate, castor oil, sesame oil, PEG 400, tween 20, tween 80.	40.02±0.83 to 99.66±1.43 nm	−26.7 to −30.6 mV	Wistar rats	Hastens wound healing process.	^107^
2.	Growth Factor (epidermal growth factor (EGF), insulin-like growth factor-I (IGF-I).	Oil-phase titration	Oleic acid, Labrasol, Transcutol.	127±4.30 nm	−24.29 mV	NH 3T mouse cells	Rapid and prolonged wound healing.	^108^
3.	Beta-glucan	Ultrasonication	Alginate, chitosan, soybean oil, span 80, tween 80.	200 nm	−38.8 mV	Human dermal fibroblast cells.	Bacterial growth inhibitory activity.	^67^
4.	Atorvastatin	Homogenization	Carboxymethyl cellulose, tween 80 and propylene glycol.	148 nm	–	Wistar rat	Improved wound healing activity.	^109^
5.	β-caryophyllene	High-speed homogenization	Span 20, tween 20, paraplast.	284.82±1.438 nm	−35.72±1.103 mV	Rat paw edema and mouse ear edema.	Superior bioadhesiveness.	^66^
6.	Curcumin	Ultrasonication	Carbopol 940, tween 20, tween 40.	56.25±0.69 nm	−20.26±0.65 mV	Rats	Exhibited thixotropic rheological behavior.	^110^
7.	Tea tree oil	Spontaneous emulsification.	Poly(caprolactone), span 80, tween 80.	198 nm	−11.5 mV	Wistar rats	Increase the biological activities of oil and protect the skin damage.	^68^

As a result, many research attempts have been made globally, prompting further efforts towards the development of nanoemulsions based hydrogels for wound healing. It has been found that treatment with the above-mentioned formulations hastens wound healing activity.

### Future perspectives and conclusion

Chronic wounds remain a major concern of the healthcare system and affect the livelihood of patients. Various drug delivery strategies for wounds are constantly being updated by several efforts carried out in different formulation avenues such as nanoemulsions, hydrogels, wafers, etc. Nanoemulsion-based wound therapy can overcome various constraints like slow healing, off-targeting, patient incompliance, and frequent dosing. Inspite of significant advantages, many sturdy challenges are still need to be overcome like cost implications, scaling up nanoemulsion production for accelerated wound healing, from the pilot level to the commercial market, etc. More toxicity evaluation on nanoemulsion-based wound healing systems might potentiate further sophisticated development. Efficient tuning of the various techniques and ingredients used in nanoemulsions production may also result in the systems having a better wound healing profile. More comprehensive understanding of the mechanism involved between nanoemulsion and wound healing will definitely allow their optimization and consequently expand enormous application aspects. Moreover, the kinetics of emulsion plays a crucial role in the production of nanoemulsions, which ultimately affect wound healing efficacy. Therefore, more investigational research efforts are required on wound healing strategy. Despite the paucity of research, 3-D bioprinting is considered as one of the most effective approaches in the vistas of skin tissue engineering and can be utilized by surgeons very effectively to develop complex organs. Another intriguing area is self-healing hydrogels, which can be printed, maintain their pre-vascularized microstructure, and function as self-healing scaffolds for wound healing. Future research should also be focused on the bioprinting of skin wound treatments on animal models.

As there are limited research advancements and patents in recent years, which demonstrates the emergence of numerous nanotherapeutics, particularly nanoemulsion systems in wound healing, indicating that much work still needs to be done toward nanotherapeutic measures in the wound healing domain. Even though there is only preliminary levels of progress in research related to the application of nanoemulsion in accordance to wound treatment, further, imperative and powerful evidence at the clinical or human level will be of great succor. Patients with chronic wounds face many challenges due to a limited understanding of wound pathophysiology, which impedes the development of advanced nano-based wound therapies. Several tailored therapies based on phenotype and genotype characteristics, in near future is possible for advanced wound treatment. Nanotechnology approaches will surely be streamlined and individualized treatment plans will also be facilitated from such platforms, resulting in major opportunities for adaptation and invention. Nanotherapeutic interventions are highly anticipated in wound healing given the emergence of various nanotechnologies, particularly multifunctional nanosystems. Nevertheless, there is a significant challenge in obtaining sufficient information on the physicochemical properties and their predicted behavior in the human body. Extensive efforts are also required to develop chronic wound therapies with site-specificity and targeting effectiveness in order to prevent undesired occurrences and interferences that could impair the biological activities of the nanosystems in the human body. Long-term studies are required to provide insight into how nanotechnology-based medicines can be used clinically. We expect that advances in innovative manufacturing and nanosystems development, as well as the knowledge of chronic wounds will lead to the development of efficient nanoemulsion-based wound healing products in the near future. Future products might take the shape of nanoemulsion loaded dressings. This could reduce the clinical-stage failure rate that many of the technologies generally faces, and to achieve quick wound healing, one should be aware of the major difficulties associated with nanoemulsion loaded wound dressings. Currently, several human studies are enrolled utilizing silver nanoparticles, meschencymal stem cell based therapies, collagen/gelatin scaffolds, 3-D printing, and so on, for wound healing. Thus, equal and necessitate development is still required to strengthen the potential of nanoemulsion in clinical wound therapy. As a result, there is also an ongoing need for improved recombinant tools and analytical techniques that will enable the clinical translation of nanoemulsion-based strategies. Although much effort has been made to use nanoemulsion-like formulations in wound healing, there is still much potential for improvement and several problems need to be fixed urgently. Thus, additional clinical studies must be conducted in future research to support the fabrication of nanoemulsions for wound healing. The development of more conceptually inventive design processes for nanoemulsion for efficient application in wound healing is envisaged in the next years. For nanoemulsion development in wound healing applications, numerous factors, including clinical translation should be taken into account. The majority of recently formulated products based on nanotechnology are now quite expensive, which restricts their clinical availability. It seems feasible and advantageous to employ randomized clinical trials to advance research into the efficacy of therapies. Moreover, a lot of research still needs to be done urgently in the area of patents related to wound healing and nanoemulsions. Wound healing both internally or externally is a complex mechanism that requires prompt action within time. Nanoemulsions have a smaller particle size and controlled release action that leads to several encouraging and attractive benefits in wound therapy such as improved skin permeation, controlled release, protection of the drug from the unfavorable environment in the body, and reduced toxicity.

The present review summarized the important wound healing aspects, wound classification, influencing factors and strategies for the improved wound healing. Nanoemulsions loaded with natural oils and their impressive role in wound healing have been described. The mechanism of nanoemulsions in wound treatment has also been clearly illustrated. Moreover, different research advancements of nanoemulsions comprising of synthetic and natural actives that aid in the wound healing process are also explained in the manuscript. Sophisticated developments for nanoemulsion-based hydrogels have also been portrayed for enhanced wound healing profile. These advancements in wound therapy have provided new hopes for patients suffering from severe chronic wounds.

## Ethical approval

Not applicable.

## Sources of funding

No funding.

## Author contributions

J.C.: conceptualization, data curation, writing-original draft preparation, writing- reviewing and editing; H.C.: conceptualization, data curation, writing-original draft preparation, writing- reviewing and editing; R.P.: data curation, writing-original draft preparation, writing- reviewing and editing; N.R.: data curation, writing-original draft preparation, writing- reviewing and editing; K.W.: data curation, writing-original draft preparation, writing-reviewing and editing; S.S.: data curation, writing-original draft preparation, writing-reviewing and editing; P.N.: data curation, writing-original draft preparation, writing-reviewing and editing; M.G.: data curation, writing-original draft preparation, writing-reviewing and editing; I.S.: data curation, writing-original draft preparation, writing-reviewing and editing; H.D.: data curation, writing-original draft preparation, writing-reviewing and editing; T.B.E.: writing-reviewing and editing, visualization, supervision.

## Conflicts of interest disclosure

Authors declare that they have no conflicts of interest.

## Research registration unique identifying number (UIN)

1. Name of the registry: Not applicable.

2. Unique Identifying number or registration ID: Not applicable.

3. Hyperlink to your specific registration (must be publicly accessible and will be checked): Not applicable.

## Guarantor

Talha Bin Emran, Department of Pharmacy, BGC Trust University Bangladesh, Chittagong 4381, Bangladesh. Tel: +880 303 356 193, fax: +880 312 550 224, Cell: +880 181 994 2214. https://orcid.org/0000-0003-3188-2272. Department of Pharmacy, Faculty of Allied Health Sciences, Daffodil International. University, Dhaka 1207, Bangladesh. E-mail: talhabmb@bgctub.ac.bd.

## Data availability statement

No specific data collected for the above manuscript.

## Provenance and peer review

Not commissioned, internally peer-reviewed.

## Acknowledgements

The authors are thankful to their parent institutions.
